# Application of Boosting Regression Trees to Preliminary Cost Estimation in Building Construction Projects

**DOI:** 10.1155/2015/149702

**Published:** 2015-08-03

**Authors:** Yoonseok Shin

**Affiliations:** Department of Plant and Architectural Engineering, Kyonggi University, Gwanggyosan-ro 154-42, Yeongtong-gu, Suwon, Gyeonggi-do 443-760, Republic of Korea

## Abstract

Among the recent data mining techniques available, the boosting approach has attracted a great deal of attention because of its effective learning algorithm and strong boundaries in terms of its generalization performance. However, the boosting approach has yet to be used in regression problems within the construction domain, including cost estimations, but has been actively utilized in other domains. Therefore, a boosting regression tree (BRT) is applied to cost estimations at the early stage of a construction project to examine the applicability of the boosting approach to a regression problem within the construction domain. To evaluate the performance of the BRT model, its performance was compared with that of a neural network (NN) model, which has been proven to have a high performance in cost estimation domains. The BRT model has shown results similar to those of NN model using 234 actual cost datasets of a building construction project. In addition, the BRT model can provide additional information such as the importance plot and structure model, which can support estimators in comprehending the decision making process. Consequently, the boosting approach has potential applicability in preliminary cost estimations in a building construction project.

## 1. Introduction

In building construction, budgeting, planning, and monitoring for compliance with the client's available budget, time, and work outstanding are important [1]. The accuracy of the construction cost estimation during the planning stage of a project is a crucial factor in helping the client and contractor with the adequate decision making and for the successful completion of the project [2–5]. However, there is a problem in that it is difficult to quickly and accurately estimate the construction costs at the early stage because the drawings and documentation are generally incomplete [6]. Machine learning approaches can be applied to alleviate this problem. Machine learning has some advantages over the human-crafted rules for data driven works, that is, accurate, automated, fast, customizable, and scalable [7].

Cost estimating approaches using a machine learning technique such as a neural network (NN) or support vector machine (SVM) have received significant attention since the early 1990s for accurately predicting the construction costs under a limited amount of project information. The NN model [1, 8–11] and the SVM model [12–16] were developed for predicting and/or estimating the construction costs. Although applying an NN to construction cost estimations has been very popular and has shown superior accuracy over other competing techniques [2, 4, 17–21], it has several disadvantages, such as a lack of self-learning and a time-consuming rule acquisition process [14]. A SVM, introduced by Vapnik [22], has attracted a great deal of attention because of its capacity for self-learning and high performance in generalization; moreover, it has shown the potential for utilization in construction cost estimations [5, 13, 14, 16, 23, 24]. However, the SVM approach requires a great deal of trial and error to determine a suitable kernel function [14]. Moreover, SVM models have a high level of algorithmic complexity and require extensive amounts of memory [25].

Among the recent machine learning techniques, the boosting approach, which was developed by Freund and Schapire [26], who also introduced the AdaBoost algorithm, has become an important application in machine learning and predicting models [27]. The boosting approach provides an effective learning algorithm and strong boundaries in terms of the generalization performance [28–31]. Compared with competing techniques used for prediction problems, the performance of the boosting approach is superior to that of both a NN [32] and a SVM [33]. It is also simple, easy to program, and has few parameters to be tuned [31, 34, 35]. Because of these advantages, the boosting approach has been actively utilized in various domains. In the construction domain, some studies have attempted to apply this approach to the classification problem (for predicting a categorical dependent variable), such as the prediction of litigation results [27] and the selection of construction methods [31, 36]. However, there have been no efforts to do so for regression problems (for predicting a continuous dependent variable), such as construction cost estimation.

In this study, the boosting regression tree (BRT) is applied to the cost estimation at the early stage of a construction project to examine the applicability of the boosting approach for a regression problem within the construction domain. The BRT in this study is based on the module of a stochastic gradient boosting tree, which was proposed by Friedman (2002) [37]. It was developed as a novel advance in data mining that extends and improves the regression tree using a stochastic gradient boosting approach. Therefore, it has advantages of not only a boosting approach but also a regression tree, that is, high interpretability, conceptual simplicity, computational efficiency, and so on. The boosting approach can especially adopt the other data mining techniques, that is, a NN and SVM, as well as decision tree, as base learner. This feature matches up to the latest trends in the field of fusion of computational intelligence techniques to develop efficient computational models for solving practical problems.

In the next section, the construction cost estimation and its relevant studies are briefly reviewed. In the third section, the theory of a BRT and a cost estimation model using a BRT are both described. In the fourth section, the cost estimation model using a BRT is applied to a dataset from an actual project of a school building construction in Korea and is compared with that of an NN and an SVM. Finally, some concluding remarks and suggestions for further study are presented.

## 2. Review of Cost Estimation Literature

Raftery [38] categorized the preliminary cost estimation system used in building construction projects into three generations. The first generation of the system was a method from the late 1950s to the late 1960s that utilized the unit-price. The second generation of the system, which was developed from the middle of the 1970s, was a statistical method using a regression analysis according to propagating personal computers. The third generation of the system is a knowledge-based artificial intelligence method from the early 1980s. However, based on the third generation, Kim [39] also separated a fourth generation based on machine learning techniques such as a NN and SVM. The author showed an outstanding performance in construction cost estimation, although much remains to be resolved, for example, the complexity of the parameter settings.

We believe that the boosting approach can be a next-generation cost estimation system at the early stage of a construction project. In the prediction problem domain, combining the predictors of several models often results in a model with improved performance. The boosting approach is one such method that has shown great promise. Empirical studies have shown that combining models using the boosting approach produces a more accurate regression model [40]. In addition, the boosting approach can be extensively applied to prediction problems using an aforementioned machine learning technique such as a NN and SVM, as well as decision trees [27]. However, the boosting approach has never been used in regression problems of the construction domain, including cost estimations, but has been actively utilized in other domains, such as remote aboveground biomass retrieval [41], air pollution prediction [42], software effort estimation [43], soil bulk density prediction [44], and Sirex noctilio prediction [45]. In this study, we examine the applicability of a BRT for estimating the costs in the construction domain.

## 3. Boosting Regression Trees

**Figure 1:**
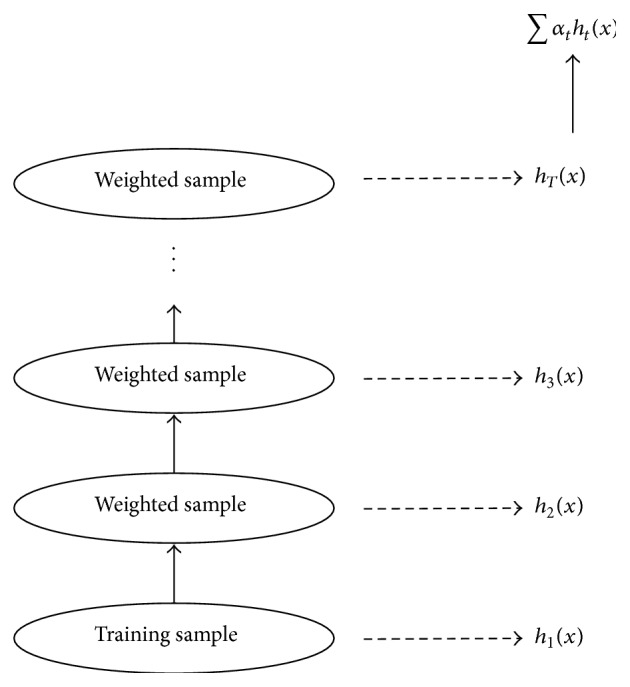
Schematic of a boosting procedure.

Because of the abundance of exploratory tools, each having its own pros and cons, a difficult problem arises in selecting the best tool. Therefore, it would be beneficial to try to combine their strengths to create an even more powerful tool. To a certain extent, this idea has been implemented in a new family of regression algorithms referred to under the general term of  “boosting.” Boosting is an ensemble learning method for improving the predictive performance of a regression procedure, such as the use of a decision tree [46]. As shown in Figure 1, the method attempts to boost the accuracy of any given learning algorithm by fitting a series of models, each having a low error rate, and then combining them into an ensemble that may achieve better performance [36, 47]. This simple strategy can result in a dramatic improvement in performance and can be understood in terms of other well-known statistical approaches, such as additive models and a maximum likelihood [48].

Stochastic gradient boosting is a novel advance to the boosting approach proposed by Friedman [37] at Stanford University. Of the previous studies [26, 49–51] related to boosting for regression problems, only Breiman [50] alludes to involving the optimization of a regression loss function as part of the boosting algorithm. Friedman [52] proposed using the connection between boosting and optimization, that is, the gradient boost algorithm. Friedman [37] then showed that a simple subsampling trick can greatly improve the predictive performance of stochastic gradient boost algorithms while simultaneously reducing their computational time.

**Figure 2:**
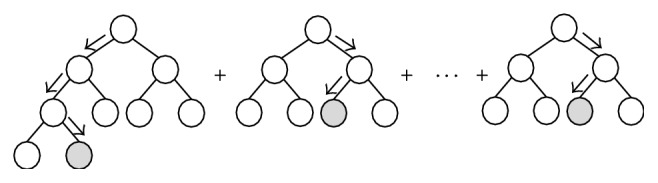
Gradient boosted decision tree ensemble.

The stochastic gradient boost algorithm proposed by Friedman [37] uses regression trees as the basis functions. Thus, this boosting regression tree (BRT) involves generating a sequence of trees, each grown on the residuals of the previous tree [46]. Prediction is accomplished by weighting the ensemble outputs of all regression trees, as shown in Figure 2 [53]. Therefore, this BRT model inherits almost all of the advantages of tree-based models, while overcoming their primary disadvantages, that is, inaccuracies [54].

In these algorithms, the BRT approximates the function *F*(*x*) as an additive expansion of the base learner (i.e., a small tree) [43]:

(1) $$\begin{array}{c} F\left(x\right)=F_{0}\left(x\right)+{\beta_{1}F}_{1}\left(x\right)+{\beta_{2}F}_{2}\left(x\right)+\cdots +{\beta_{m}F}_{m}\left(x\right). \end{array}$$

A single base learner does not make sufficient prediction using the training data, even when the best training data are used. It can boost the prediction performance using a series of base learners with the lowest residuals.

Technically, BRT employs an iterative algorithm, where, at each iteration *m*, a new regression tree *h*(*x*; {*R*_*lm*_}_*l*_^*L*^) partitions the *x*-space into *L*-disjoint regions {*R*_*lm*_}_*l*_^*L*^ and predicts a separate constant value in each one [54]:

(2) $$\begin{array}{c} h\left(x;{\left\{R_{lm}\right\}}_{l}^{L}\right)=\overset{L}{\underset{l-1}{\sum }}{\overset{-}{y}}_{lm}\;|\;\left(x\in R_{lm}\right). \end{array}$$

Here ${\overset{-}{y}}_{lm}={mean}_{x_{i}\in R_{lm}}({\tilde{y}}_{im})$ is the mean of pseudo-residuals (3) in each region *R*_*lm*_ induced at the *m*th iteration [37, 54]:

(3) $$\begin{array}{c} {\tilde{y}}_{im}=-{\left[\frac{\partial \Psi \left(y_{i},F\left(x_{i}\right)\right)}{\partial F\left(x_{i}\right)}\right]}_{F\left(x\right)=F_{m-1}\left(x\right)}. \end{array}$$

The current approximation *F*_*m*−1_(*x*) is then separately updated in each corresponding region [37, 54]:

(4) $$\begin{array}{c} F_{m}\left(x\right)=F_{m-1}\left(x\right)+\upsilon \cdot \gamma_{lm}\;|\;\left(x\in R_{lm}\right), \end{array}$$

where

(5) $$\begin{array}{c} \gamma_{lm}=\underset{\gamma }{\arg\;\min}\overset{}{\underset{x_{i}\in R_{Im}}{\sum }}\Psi \left(y_{i},F_{m-1}\left(x_{i}\right)+\gamma \right). \end{array}$$

The “shrinkage” parameter *υ* controls the learning rate of the procedure.

This leads to the following BRT algorithm for generalized boosting of regression trees [37].

(1) Initialize *F*(*x*), *F*_0_(*x*) = argmin_*Υ*_⁡∑_*i*−1_^*N*^Ψ(*y*_*i*_, *γ*).
(2) For *m* = 1 to *M* do
(3) Select a subset randomly from the full training dataset,
  (6) $$\begin{array}{c} {\left\{\pi \left(i\right)\right\}}_{l}^{N}=rand\_perm{\left\{i\right\}}_{l}^{N}. \end{array}$$
(4) Fit the base learner,
  (7) $$\begin{array}{c} {\tilde{y}}_{\pi \left(i\right)m}=-{\left[\frac{\partial \Psi \left(y_{\pi \left(i\right)m,},F\left(x_{\left(i\right)}\right)\right)}{\partial F\left(x\left(i\right)\right)}\right]}_{F\left(x\right)=F_{m-1}\left(x\right)},\;\;i=1,\tilde{N}\text{.} \end{array}$$
(5) Compute the model update for the current iteration,
  (8) $$\begin{array}{c} {\left\{R_{lm}\right\}}_{I}^{L}=L-terminal\;node\;tree\left({\left\{{\tilde{y}}_{\pi \left(i\right)m,}x_{\pi \left(i\right)}\right\}}_{l}^{\tilde{N}}\right). \end{array}$$
(6) Choose a gradient descent step size as,
  (9) $$\begin{array}{c} \gamma_{lm}=\underset{\gamma }{arg\;min}\overset{}{\underset{x_{\left(i\right)}\in R_{lm}}{\sum }}\Psi \left(y_{\pi \left(i\right)},F_{m-1}\left(x_{\pi \left(i\right)}\right)+\gamma \right). \end{array}$$
(7) Update the estimate of *F*(*x*) as,
  (10) $$\begin{array}{c} F_{m}\left(x\right)=F_{m-1}\left(x\right)+\upsilon \cdot \gamma_{lm}\;|\;\left(x\in R_{lm}\right). \end{array}$$
(8) end For.

**Figure 3:**
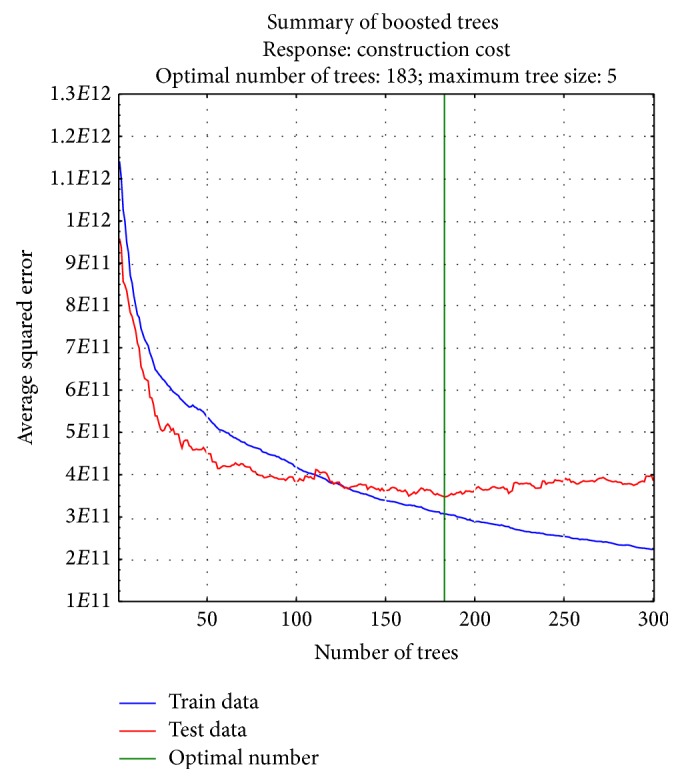
Training results of BRT.

There are specific algorithms for several loss criteria including least squares: *ψ*(*y*, *F*) = (*y* − *F*)^2^, least-absolute deviation: *ψ*(*y*, *F*) = |*y* − *F*|, and Huber-*M*: *ψ*(*y*, *F*) = (*y* − *F*)^2^∣(|*y* − *F*|≼*δ*) + 2*δ*(|*y* − *F*| − *δ*/2)∣(|*y* − *F*| > *δ*) [37]. The BRT applied in this study adopts the least squares for loss criteria as shown in Figure 3.

## 4. Application

### 4.1. Determining Factors Affecting Construction Cost Estimation

In general, the estimation accuracy in a building project is correlated with the amount of project information available regarding the building size, location, number of stories, and so forth [55]. In this study, the factors used for estimating the construction costs are determined in two steps. First, a list of factors affecting the preliminary cost estimation was made by reviewing previous studies [2, 3, 8, 12, 14, 20, 23, 55, 56]. Lastly, appropriate factors were selected from this list by interviewing practitioners who are highly experienced in construction cost estimation in Korea. Consequently, nine factors (i.e., input variables) were selected for this study, as shown in Table 1.

### 4.2. Data Collection

**Figure 4:**
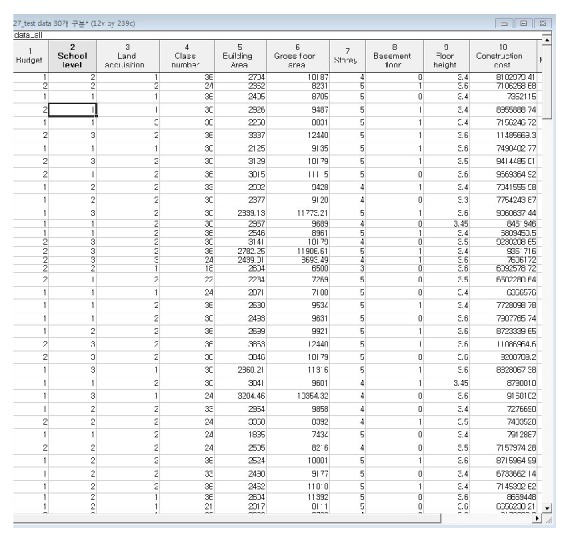
Fragment of cost dataset.

Data were collected from 234 completed school building projects executed by general contractors from 2004 to 2007 in Gyeonggi Province, Korea. These cost data were only the direct costs of different school buildings, such as elementary, middle, and high schools, without a markup as shown in Figure 4. According to the construction year, the total construction costs were converted using the Korean building cost index (BCI); that is, the collected cost data were multiplied by the BCI of the base year of 2005 (BCI = 1.00). The collected cost data of 217 school buildings were randomly divided into 30 test datasets and 204 training datasets.

### 4.3. Applying BRT to Construction Cost Estimation

In this study, the construction cost estimation model using a BRT was tested through application to real building construction projects. The construction costs were estimated using the BRT as follows. (1) The regression function $\hat{F}\left(x\right)$ was trained using training data. In the dataset, the budget, school levels, gross floor area, and so on were allocated to each *x*_*i*_ of the training set. Each result, that is, the actual cost, was allocated to *y*_*i*_. (2) After the training was completed according to the parameters such as the learning (shrinkage) rate, the number of additive trees, and the maximum and minimum number of levels, the series of trees $\hat{F}\left(x\right)$ which maps *x* to *y* of training data set (*y*_*i*_, *x*_*i*_) with minimized loss function Ψ(*y*_*i*_, *F*(*x*_*i*_)) was found. (3) The expected value of  $\hat{F}\left(x\right)$, that is, the expected cost, was calculated for a new test dataset (*y*_*j*_, *x*_*j*_).

**Figure 5:**
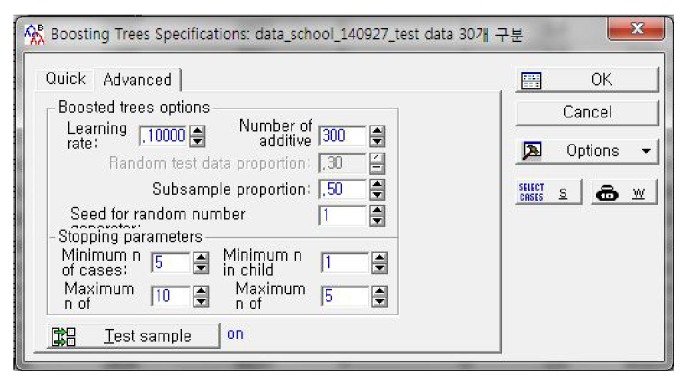
Parameter setting for BRT.

The construction cost estimation model proposed in this study was constructed using “STATISTICA Release 7.” STATISTICA employs an implementation method usually referred to as a stochastic gradient boosting tree by Friedman (2002, 2001) [37, 52], also known as TreeNet (Salford Systems, Inc.) or MART (Jerill, Inc.). In this software, a stochastic gradient booting tree is used for regression problems to predict a continuous dependent variable [57]. To operate a boosting procedure in STATISTICA, the parameter settings, that is, the learning rate, the number of additive trees, the proportion of subsampling, and so forth, are required. Firstly, the learning rate was set as 0.1. It was found that small values, that is, values under 0.1, lead to much better results in terms of the prediction error [52]. We empirically obtained the other parameters, which are shown in Figure 5. As a result, the training result of the BRT showed that the optimal number of additive trees is 183 and the maximum size of tree is 5, as shown in Figure 3.

### 4.4. Performance Evaluation

In general, the cost estimation performance can be measured based on the relationship between the estimated and actual costs [56]. In this study, the performance was measured using the Mean Absolute Error Rates (MAERs), which were calculated using

(11) $$\begin{array}{c} MAERs=\frac{\left(\sum \left|\left(\left(C_{e}-C_{a}\right)/C_{a}\right)\times 100\right|\right)}{n}, \end{array}$$

where *C*_*e*_ is the estimated construction costs by model application, *C*_*a*_ is the actual construction costs collected, and *n* is the number of test datasets.

To verify the performance of the BRT model, the same cases were applied to a model based on a NN and the results compared. We chose the NN model because it showed a superior performance in terms of cost estimation accuracy in previous studies [2, 5, 14]. “STATISTICA Release 7” was also used to construct the NN model in this study. To construct a model using a NN, the optimal parameters have to be selected beforehand, that is, the number of hidden neurons, the momentum, and the learning rate for the NN. Herein, we determined the values from repeated experiments.

## 5. Results and Discussion

### 5.1. Results of Evaluation

The results from the 30 test datasets using a BRT and a NN are summarized in Tables 2 and 3. The results from the BRT model had MAERs of 5.80 with 20% of the estimates within 2.5% of the actual error rate, while 80% were within 10%. The NN model had MAERs of 6.05 with 10% of the estimates within 2.5% of the actual error rate, while 93.3% were within 10%. In addition, the standard deviations of the NN and BRT models are 3.192 and 3.980, respectively, as shown in Table 4.

The MAERs of two results were then compared using a *t*-test analysis. The MAERs of the two results are statistically similar, although there are differences between them. As the null hypothesis, the MAERs of the two results are all equal (*H*_0_ : *u*_*D*_ = 0). The *t*-value is 0.263 and the *P* value is 0.793 (>0.05). Thus, the null hypothesis is accepted. This analysis shows that the MAERs of the two results are statistically similar.

**Figure 6:**
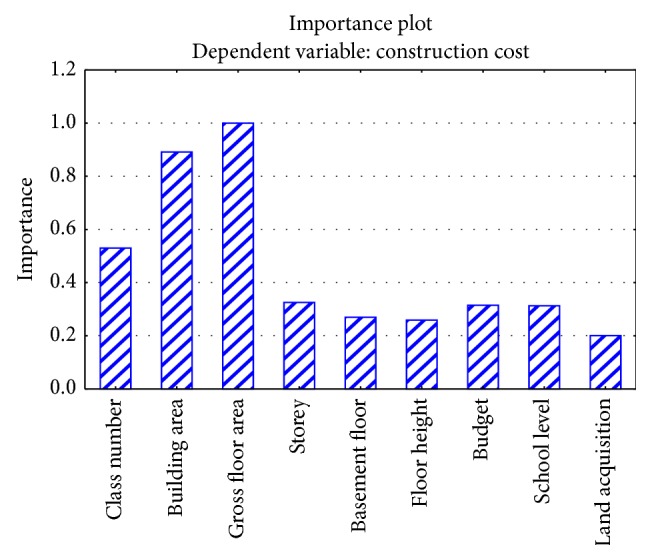
Importance plot of dependent variables.

**Figure 7:**
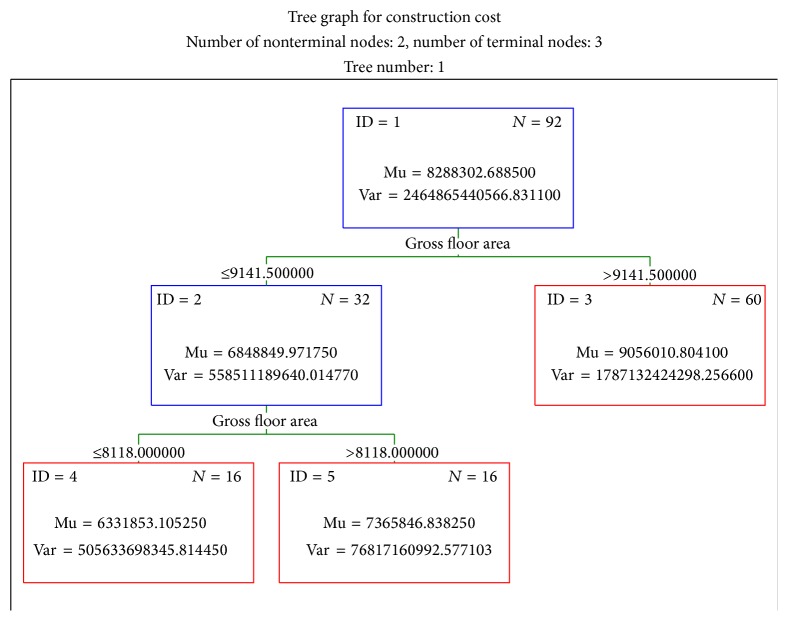
An example of structure model.

The BRT model provided comprehensible information regarding the new cases to be predicted, which is an advantage inherent to a decision tree. Initially, the importance of each dependent variable to cost estimation was provided, as shown in Figure 6. These values indicate the importance of each variable for the construction cost estimation in the model. Finally, the tree structures in the model were provided as shown in Figure 7. This shows the estimation rules, such as the applied variables and their influence on the proposed model. Thus, an intuitive understanding of the whole structure of the model is possible.

### 5.2. Discussion of Results

This study was conducted using 234 school building construction projects. In addition, 30 of these projects were used for testing. In terms of the estimation accuracy, the BRT model showed slightly better results than the NN model, with MAERs of 5.80 and 6.05, respectively. In terms of the construction cost estimation, it is difficult to conclude that the performance of the BRT model is superior to that of the NN model because the gap between the two is not statistically different. However, even the similar performance of the BRT model is notable because the NN model has proven its superior performance in terms of cost estimation accuracy in previous studies. Similarly, in predicting the software project effort, Elish [43] compared the estimation accuracy of neural network, linear regression, support vector regression (SVR), and BRT. Consequently, BRT outperformed the other techniques in terms of the estimation performance that has been also achieved by SVR. These results mean that the BRT has remarkable performance in regression problem as well as classification one. Moreover, the BRT model provided additional information, that is, an importance plot and structure model, which helps the estimator comprehend the decision making process intuitively.

Consequently, these results reveal that a BRT, which is a new AI approach in the field of construction, has potential applicability in preliminary cost estimations. It can assist estimators in avoiding serious errors in predicting the construction costs when only limited information is available during the early stages of a building construction project. Moreover, a BRT has a large utilization possibility because the boosting approach can employ existing AI techniques such as a NN and SVM, along with decision trees, as base learners during the boosting procedure.

## 6. Conclusion

This study applied a BRT to construction cost estimation, that is, the regression problem, to examine the applicability of the boosting approach to a regression problem in the construction domain. To evaluate the performance of the BRT model, its performance was compared with that of an NN model, which had previously proven its high performance capability in the cost estimation domains. The BRT model showed similar results when using 234 actual cost datasets of a building construction project in Korea. Moreover, the BRT model can provide additional information regarding the variables to support estimators in comprehending the decision making process. These results demonstrated that the BRT has dual advantages of boosting and decision trees. The boosting approach has great potential to be a leading technique in next generation construction cost estimation systems.

In this study, an examination using a relatively small dataset and number of variables was carried out on the performance of a BRT for construction cost estimation. Although both models performed satisfactorily, further detailed experiments and analyses regarding the quality of the collected data are necessary to utilize the proposed model for an actual project.

## Figures and Tables

**Table 1:** Factors in construction cost estimation.

Description	Min.	Max	Average	Remark
Input				
Budget		(1) BTL		Nominal
		(2) National finance		
School levels		(1) Elementary		Nominal
		(2) Middle		
		(3) High		
Land acquisition		(1) Existing		Nominal
		(2) Building lots		
		(3) Green belts		
Class number	12	48	31	Numerical
Building area (m^2^)	1,204	3,863	2,694	Numerical
Gross floor area (m^2^)	4,925	12,710	9,656	Numerical
Storey	3	7	4.7	Numerical
Basement floor (storey)	0	2	0.5	Numerical
Floor Height (m)	3.3	3.6	3.5	Numerical
Output				
Total construction cost (thousand KRW)	4,334,369	14,344,867	8,288,008	Numerical

**Table 2:** Summary of results by estimation model.

Error rate (%)	NN		BRT	
	Fre. (%)	Cum. (%)	Fre. (%)	Cum. (%)
0.0–2.5	3 (10.0)	3 (10.0)	6 (20.0)	6 (20.0)
2.5–5.0	11 (36.7)	14 (46.7)	10 (33.3)	16 (53.3)
5.0–7.5	6 (20.0)	20 (66.7)	6 (20.0)	22 (73.3)
7.5–10.0	8 (26.7)	28 (93.3)	2 (6.7)	24 (80.0)
10.0–12.5	1 (3.3)	29 (96.7)	3 (10.0)	27 (90.0)
12.5–15.0	1 (3.3)	30 (100)	2 (6.7)	29 (96.7)
15.0–17.5	0 (0)	30 (100)	1 (3.3)	30 (100)

MAERs	6.05	—	5.80	—

**Table 3:** Cost estimation results of each test set.

Number	Historical cost (1,000 KRW)	Neural networks		Boosting regression tree	
		Predicted cost (1,000 KRW)	Error rate (%)	Predicted cost (1,000 KRW)	Error rate (%)
1	6,809,450	7,704,034	13.14	7,206,795	5.84
2	9,351,716	10,015,906	7.10	9,805,656	4.85
3	6,656,230	7,251,317	8.94	6,322,112	5.02
4	7,119,470	7,128,513	0.13	7,418,373	4.20
5	7,304,747	7,978,990	9.23	7,349,178	0.61
6	9,729,392	9,516,946	2.18	9,259,162	4.83
7	10,801,826	9,817,999	9.11	9,682,119	10.37
8	7,944,318	7,246,763	8.78	7,136,773	10.17
9	10,879,004	10,136,431	6.83	10,572,777	2.81
10	7,552,814	7,764,300	2.80	7,683,295	1.73
11	8,845,099	8,558,536	3.24	8,370,497	5.37
12	10,690,800	10,001,503	6.45	10,015,284	6.32
13	8,694,721	8,258,452	5.02	8,446,796	2.85
14	6,582,636	6,810,406	3.46	6,954,507	5.65
15	7,583,680	8,312,216	9.61	8,194,292	8.05
16	7,099,220	7,955,966	12.07	8,292,381	16.81
17	8,145,147	8,604,444	5.64	8,522,009	4.63
18	8,652,810	7,853,765	9.23	8,270,169	4.42
19	10,527,278	10,040,039	4.63	9,611,194	8.70
20	6,679,924	6,467,344	3.18	7,397,923	10.75
21	8,383,830	9,203,887	9.78	8,487,286	1.23
22	7,298,932	8,018,225	9.85	8,294,895	13.65
23	7,505,428	7,749,053	3.25	7,967,265	6.15
24	7,710,921	7,622,053	1.15	7,795,563	1.10
25	6,196,652	6,503,022	4.94	5,940,634	4.13
26	8,897,861	8,554,455	3.86	8,714,123	2.06
27	7,840,787	8,535,617	8.86	8,863,975	13.05
28	8,023,067	7,666,898	4.44	6,900,068	14.00
29	7,495,213	7,270,806	2.99	7,695,613	2.67
30	7,653,005	8,003,292	4.58	7,775,139	1.60

MAERs			6.05		5.80

**Table 4:** Descriptive analysis of error rate estimation.

	MAERs	Std, deviation	Std, error	95% confidence interval of the MAERs	
				Lower	Upper
NN	6.045	3.192	0.583	2.542	4.291
BRT	5.800	3.980	0.727	3.170	5.351
